# Change in inhaled corticosteroid treatment and COPD exacerbations: an analysis of real-world data from the KOLD/KOCOSS cohorts

**DOI:** 10.1186/s12931-019-1029-7

**Published:** 2019-03-28

**Authors:** Se Hee Lee, Ji-Hyun Lee, Ho Il Yoon, Hye Yun Park, Tae-Hyung Kim, Kwang Ha Yoo, Yeon-Mok Oh, Ki Suk Jung, Sang-Do Lee, Sei Won Lee

**Affiliations:** 1 Department of Pulmonary and Critical Care Medicine, Asan Medical Center, University of Ulsan College of Medicine, Seoul, Republic of Korea; 2 Department of Internal Medicine, CHA Bundang Medical Center, CHA University, Seongnam, Republic of Korea; 3 Division of Pulmonary and Critical Care Medicine, Department of Internal Medicine, Seoul National University College of Medicine, Seoul National University Bundang Hospital, Seongnam, Republic of Korea; 4 Division of Pulmonary and Critical Care Medicine, Department of Medicine, Samsung Medical Center, Sungkyunkwan University School of Medicine, Seoul, Republic of Korea; 5 Division of Pulmonary and Critical Care Medicine, Hanyang University Guri Hospital, Hanyang University College of Medicine, Guri, Republic of Korea; 6 Division of Pulmonary, Allergy and Critical Care Medicine, Department of Internal Medicine, Konkuk University School of Medicine, Seoul, Republic of Korea; 7 Division of Pulmonary, Allergy and Critical Care Medicine, Hallym University Sacred Heart Hospital, Hallym University Medical School, Anyang, Republic of Korea

**Keywords:** Bronchitis, chronic, Bronchodilator agents, Eosinophils, Glucocorticoids, Chronic obstructive pulmonary disease

## Abstract

**Background:**

This cohort study of patients with chronic obstructive pulmonary disease (COPD) was performed to evaluate the status of inhaled corticosteroid (ICS) prescriptions following the 2017 revision of the Global Initiative for Chronic Obstructive Lung Disease guidelines.

**Methods:**

A total of 1144 patients from the Korean Obstructive Lung Disease and Korea Chronic Obstructive Pulmonary Disorders Subgroup Study cohorts, with final follow-up visits completed between 2017 and 2018, were analyzed. Features indicative of ICS usage were as follows: a history of asthma, blood eosinophils of ≥300 cells/μl, or ≥ 2 exacerbations in the year prior to enrollment. Among baseline ICS users, we compared annual total and severe exacerbation rates, based on ICS continuation or withdrawal.

**Results:**

ICS-containing regimens were prescribed to 46.3% of the enrolled of patients in 2014; this decreased to 38.8% in 2017, and long-acting dual bronchodilators were used instead. Among ICS users in 2017, 47.5% did not exhibit features indicative of ICS usage; 478 used ICS at baseline, and ICS was withdrawn in 77 (16.1%) during the study period. The proportion of patients with asthma and the baseline annual exacerbation rate were greater in the ICS withdrawal groinup than in the ICS continued group (56.6% vs. 41%, *p* = 0.01; 0.79 vs. 0.53, *p* < 0.001). Annual exacerbation rates during the follow-up period were similar between the ICS-withdrawal and ICS -continued groups (0.48 vs. 0.47, *p* = 0.84); however, former exhibited a significantly higher rate of severe exacerbation (0.22 vs. 0.12, *p* = 0.03).

**Conclusions:**

Prescriptions of ICS to treat COPD decreased with increased use of long-acting dual bronchodilators. ICS withdrawal might impact severe exacerbation; the potential risks and benefits of withdrawing ICS should therefore be considered based on patients’ characteristics.

## Background

Chronic obstructive pulmonary disease (COPD) is a major global health concern: with a prevalence of 5–25% in adults, COPD is the fourth leading cause of death worldwide [1, 2]. Economic burden is also an important issue. Pharmacotherapy and exacerbation management are the primary drivers of associated medical costs [3]. Inhalers are keys to pharmacotherapy in COPD; thus, appropriate inhaler use can improve various aspects of medical costs by reducing unnecessary prescriptions and episodes of exacerbation.

Because of the chronic inflammatory nature of COPD, inhaled corticosteroids (ICS) may purportedly improve clinical outcomes of patients with COPD. Early observational studies on ICS have demonstrated that ICS significantly attenuated mortality rates [4–6]. These favorable outcomes prompted the performance of a large randomized trial; however, the only found an insignificant association with ICS and mortality [7]. ICS also failed to definitively improve lung-function outcomes [8, 9]. Prior evidence thus indicates that the effect of ICS in COPD treatment is limited to the reduction of the exacerbation and improvement of symptoms in only a portion of a patients [7, 9–14]. It should also be noted that ICS involves potential harm: prolonged use of ICS is ostensibly associated with increased risks of severe pneumonia [15–17], tuberculosis [18], oral candidiasis, bruising, and bone fractures [19].

Recently, extensive studies have demonstrated the efficacy of combined long-acting bronchodilators in the treatment of COPD [20]. Newly developed therapeutic combinations, including that of long acting muscarinic antagonist (LAMA) and long acting beta2 agonist (LABA), have achieved better outcomes than LAMA monotherapy and even combination including ICS and LABA [21–23]. This resulted in major changes to the recommended pharmacologic treatment of stable COPD in the 2017 revision of the Global Initiative for Chronic Obstructive Lung Disease (GOLD) guidelines [24]. In addition, trials have identified subsets of patients with COPD for whom ICS usage is beneficial: patients with asthma-COPD overlap syndrome (ACO), and those who experience frequent exacerbations despite the use of bronchodilators [25]. Many experts suggest that ICS can be safely withdrawn if a patient showed no previous exacerbations or asthma [26]. However, in clinical practice, ICS remains widely overused without sufficient consideration of its risks and benefits [27].

The present cohort study of patients with COPD aimed to evaluate the current status of ICS prescriptions and to inform directions of improvement in the care of patients with COPD. We 1) measured annual trends of administering inhaler therapy to patients with COPD, 2) evaluated ICS prescriptions based on clinical situations, 3) identified possible candidates among ICS users for ICS withdrawal, and 4) assessed changes in the annual frequency of exacerbation based on the continuation of ICS.

## Materials and methods

### Study population

This non-interventional cohort study reflected the real-world use of ICS and bronchodilators by analyzing data from three large multicenter COPD cohorts in Korea: the Korean Obstructive Lung Disease (KOLD) 1 and 2 [28], as well as the Korea Chronic Obstructive Pulmonary Disorders Subgroup Study (KOCOSS) [29]. Each cohort was constructed with the approval of the institutional review board of each participating center and written informed consent from all enrolled patients. Patients were prospectively recruited from the pulmonary clinics of more than 30 different hospitals in South Korea. Detailed inclusion and exclusion criteria of each cohort study are presented in e-Table 1. All three cohorts included patients with persistent airflow limitation that was not fully reversible by ATS criteria (post-bronchodilator [BD] forced expiratory volume in 1 s (FEV_1_)/ forced vital capacity (FVC) < 0.7; the KOLD 1 cohort used pre-BD FEV_1_/FVC < 0.7). Patients ever diagnosed with asthma from a physician were considered to have asthma and they were eligible for enrollment if the inclusion and exclusion criteria were met. To assess annual inhaler changes since 2014, only patients whose final follow-up visits were completed in 2017 or 2018 were included.

**Table 1:** Baseline Characteristics

	Total (*n* = 1144)	ICS User (*n* = 478)	ICS nonuser (*n* = 560)	*P*-value
Age	68.1 ± 7.9	68.2 ± 7.9	68.2 ± 7.9	0.26
Male	1057(92.4)	439(91.8)	520(92.9)	0.13
Smoking status				0.80
Current smoker	348(30.4)	147(30.8)	168(30)	
Ex-smoker	700(61.2)	295(61.7)	340(60.7)	
Non-smoker^a^	80(7.0)	31(6.5)	45(8.0)	
Pack-years	42.5 ± 25.1	43.2 ± 25.3	41.3 ± 24.3	0.26
BMI	23.4 ± 10.3	23.0 ± 3.4	23.0 ± 14.4	0.78
SGRQ	28.6 ± 17.0	33.4 ± 18.7	25.9 ± 15.5	< 0.01
CAT	14.2 ± 7.9	16.2 ± 7.9	12.9 ± 7.5	< 0.01
mMRC	1.4 ± 0.9	1.6 ± 0.9	1.2 ± 0.9	< 0.01
FVC measured, L	3.21 ± 0.82	3.03 ± 0.79	3.30 ± 0.79	< 0.01
Post-BD FEV_1_, L	1.67 ± 0.58	1.49 ± 0.54	1.75 ± 0.55	< 0.01
Post-BD FEV_1_, % predicted	61.37 ± 19.2	55.86 ± 19.12	63.38 ± 16.90	< 0.01
Post-BD FEV_1_/FVC ratio	50.88 ± 12.07	47.91 ± 12.25	52.3 ± 11.39	< 0.01
Bronchodilator response				0.003
None	968(84.8)	392(82)	486(87.3)	
BDR > 12% and 200 ml (FEV_1_)	144(12.6)	66(13.8)	65(11.7)	
BDR > 15% and 400 ml (FEV_1_) (Spanish ACO)	29(2.5)	20(4.2)	6(1.1)	
DL_co_, % predicted	75.36 ± 30.32	73.92 ± 23.99	75.52 ± 36.12	0.45
Asthma	382(33.4)	205(42.9)	166(29.6)	< 0.001
Serum eosinophils cells/μl^b^	179(102–302)	179(108-328)	178(97–280)	0.16
Eosinophils > 300 cells/μl n (%)	214(18.7)	99(20.7)	95(17.0)	0.038
Exacerbation frequency, events/person-year	0.44 ± 1.61	0.60 ± 1.65	0.33 ± 1.64	< 0.01
0	909(81.2)	354(75.2)	470(85.0)	< 0.001
1	115(10.3)	57(12.1)	51(9.2)	
≥ 2	96(8.6)	60(12.7)	32(5.8)	
GOLD group				< 0.01
A	293(25.6)	76(15.9)	179(32.0)	
B	680(59.4)	309(64.6)	322(57.5)	
C	26(2.3)	8(1.7)	15(2.7)	
D	115(10.1)	77(16.1)	36(6.4)	

Continuous data are presented as mean ± SD or median (interquartile range). Categorical variables as No. (%) unless otherwise indicated. *ACO* asthma and COPD overlap, *BD* bronchodilator, *BDR* bronchodilator response, *BMI* body mass index, *CAT* COPD assessment test, *FEV*_*1*_ forced expiratory volume in 1 s, *FVC* forced vital capacity (FVC), *ICS* inhaled corticosteroid, *mMRC* modified Medical Research Council dyspnea scale, *SGRQ* St. George’s respiratory questionnaire

^a^Among non-smokers, 61 patients had history of pulmonary disease; 40 (65.6%), asthma; 34 (55.7%), pulmonary tuberculosis; 18 (29.5%), measles; 2 (3%), pertussis; and 15 (24.5%) had pneumonia

^b^n = 853

### Study variables

The following baseline data were collected at the time of enrollment: demographics, smoking habits, body mass index (BMI), responses to St. George’s respiratory questionnaire (SGRQ), symptom scores from the COPD assessment test (CAT) and modified Medical Research Council (mMRC) dyspnea scale, history of asthma, and number of exacerbations during the year prior to enrollment. Spirometry results, including FEV_1_, FVC, and the diffusing capacity were investigated. Two criteria for the bronchodilator response were used to estimate the number of patients with reversibility: 1) the GOLD criteria and 2) the Spanish definition of ACO [30]. Patients were classified in accordance with the 2017 GOLD guidelines [24].

Any patient using ICS-containing regimens, were defined as ICS users. Others who were using non-ICS-containing regimens, including LAMA, LABA, or LAMA/LABA, were defined as ICS nonusers. In this study, features indicative of ICS usage were defined as follows: history of asthma, serum eosinophil count of ≥300 cells/μl and ≥ 2 exacerbations in the year prior to enrollment [24, 25].,

Patients who were using ICS at the time of enrollment (for patients who were enrolled after 2014) or in 2014 (for patients who enrolled before 2014) were classified as baseline ICS users. Exacerbation was defined as symptomatic deterioration requiring additional short-course treatment of antibiotics or systemic steroids with an unexpected visit to the clinic or ER, or hospitalization. Severe exacerbation was defined as an exacerbation event resulting in admission or an ER visit.

Time to first exacerbation and the annual exacerbation rates during the observation period were compared between the ICS withdrawal and continued groups. The observation period of the former was defined as the time from ICS withdrawal to the last follow up. The number of exacerbations after ICS withdrawal were analyzed for the comparison of the annual exacerbation rate.

### Statistics

Baseline characteristic data are expressed as mean ± standard deviation for continuous variables, which were analyzed using the t-test or Mann-Whitney U test. For categorical data, Fisher’s exact test was used. Descriptive analysis was used for assessing annual changes in inhalers.

Times to first exacerbation and severe exacerbation were compared with Kaplan-Meier estimates and the log-rank test. The frequencies of total and severe exacerbation during the observation period were analyzed using a generalized linear model, with the assumption of a negative binomial distribution, and were expressed as the exacerbation rate (per person-year). Other risk factors and confounders, such as baseline exacerbation frequency, post BD FEV_1_% predicted, CAT score, age, and BMI, were further adjusted using a negative binomial regression model to estimate the impact of ICS withdrawal on exacerbation frequency. All data were analyzed using SPSS software (version 24.0; IBM Corp., Armonk, NY).

## Results

### Participants

**Fig. 1:**
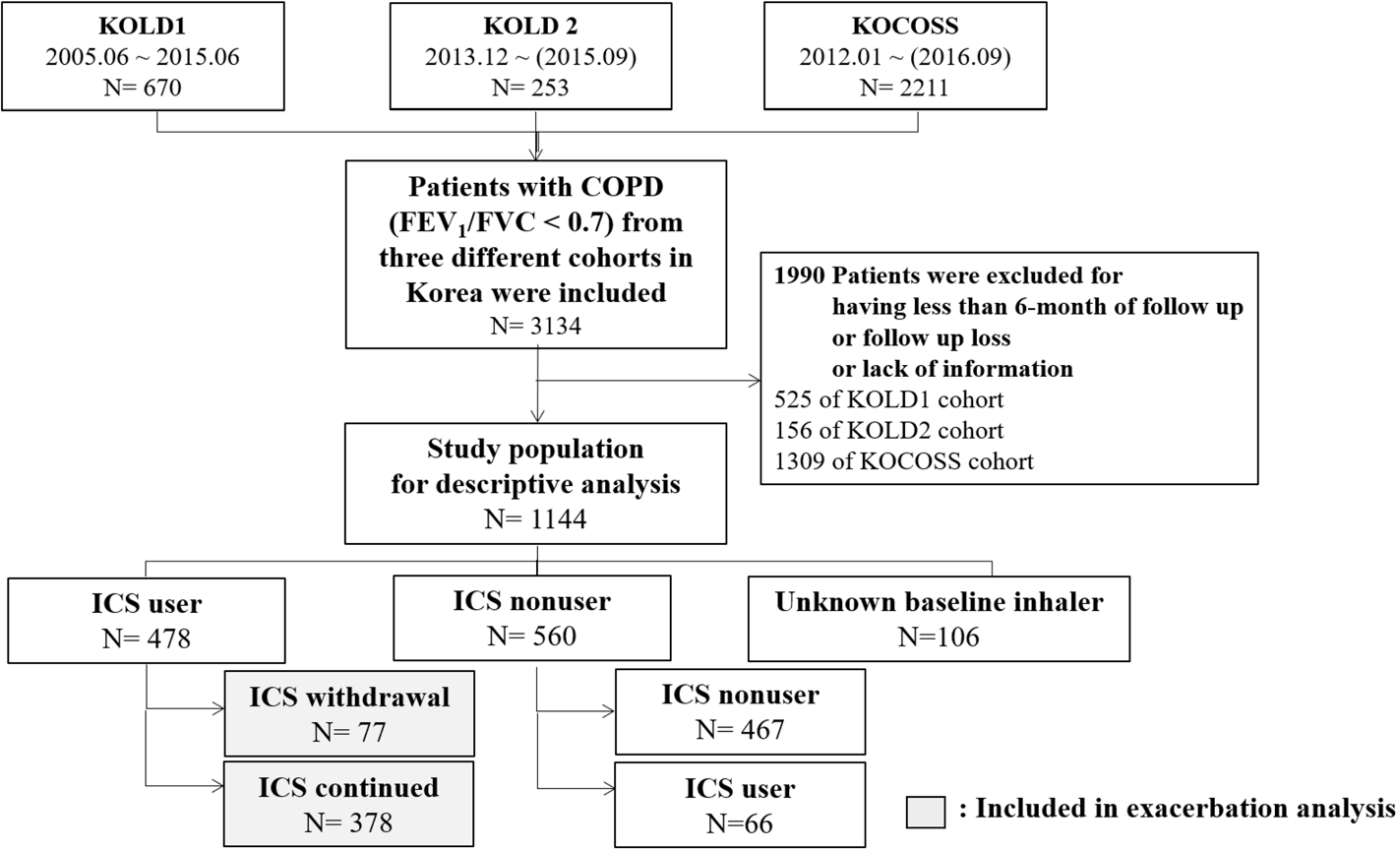
Schematic illustration of enrolled participants

Through March 1st 2018, 3134 patients who had completed their follow-ups in 2017 and 2018 were enrolled from the three different cohorts (Fig. 1). Among these patients, 1144 patients were eligible for the study. 478 patients (41.7%) were ICS users, and 560 patients (48.9%) were ICS nonusers. Among the baseline ICS users, 77 patients (16%) underwent ICS withdrawal during the follow-up period.

Demographics and baseline characteristic of the study populations are illustrated in Table 1. The proportion of patients with asthma was higher in the ICS user group. Mean exacerbation frequency per year was higher in the ICS user group than in the ICS nonuser group (0.6 vs 0.33, *p* < 0.01). The ICS user group featured a higher proportion of patients with GOLD groups B and D.

### Inhaler usage

**Fig. 2:**
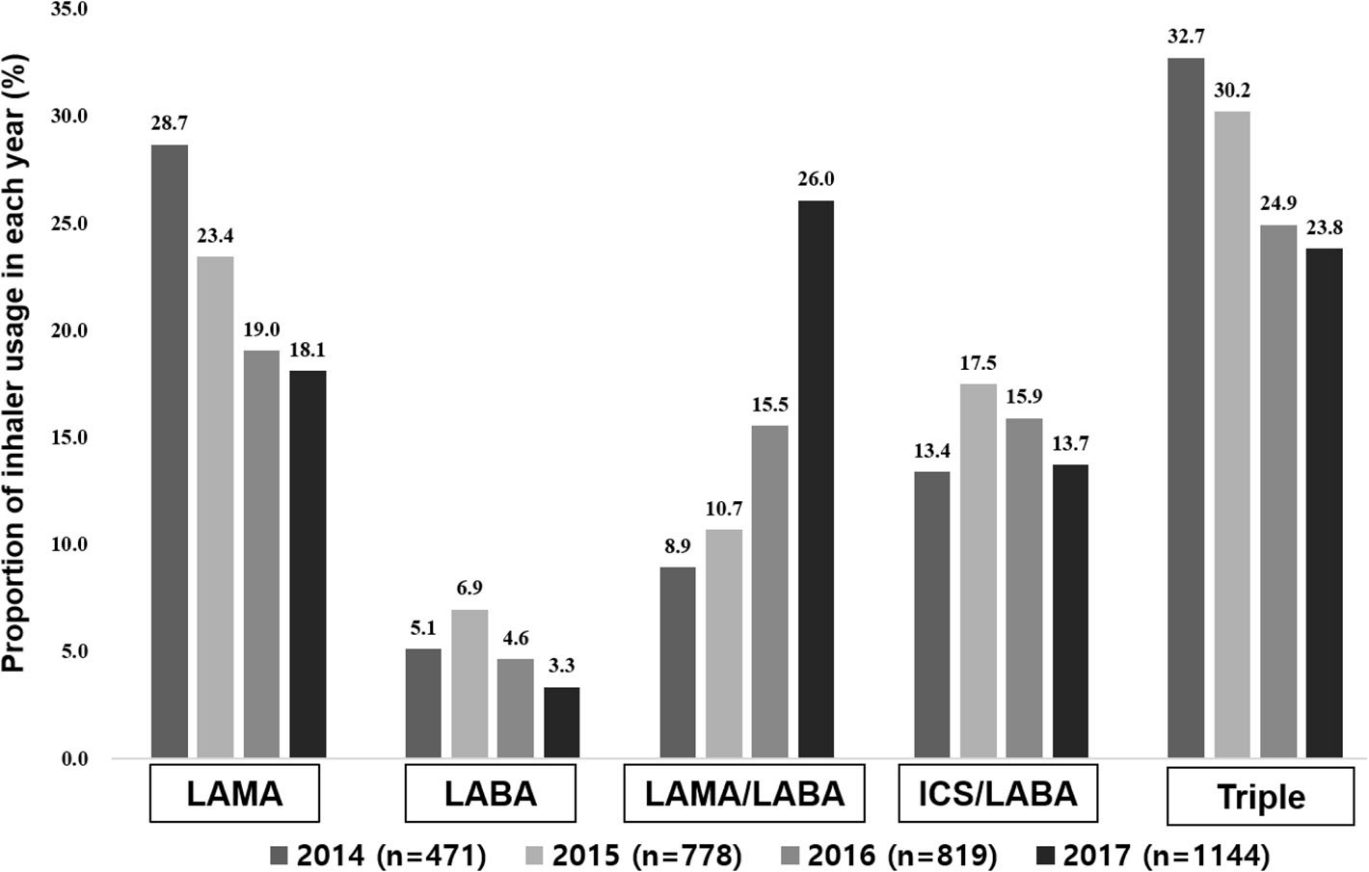
Annual changes in inhaler usage. The medication possession ratio was defined such that inhalers used for more than 6 months were regarded as representative bronchodilators for each year. ICS = Inhaled corticosteroid; LAMA = long acting muscarinic antagonist; LABA = long acting beta2 agonist

Annual inhaler usage changes are described in Fig. 2. In 2014, approximately half of the patients (46.3%) were ICS users. Triple therapy was the most frequently used regimen, but LAMA monotherapy and ICS-containing regimens were also common.

Since 2016, the percentage of patients using ICS-containing regimens—both ICS/LABA and triple therapy—decreased, while the percentage of LAMA/LABA users increased. In 2017, the LAMA/LABA combination regimen was the most frequently used regimen, followed respectively by triple therapy and LAMA monotherapy. Meanwhile, 38.8% of patients with COPD remained as ICS users in 2017.

**Fig. 3:**
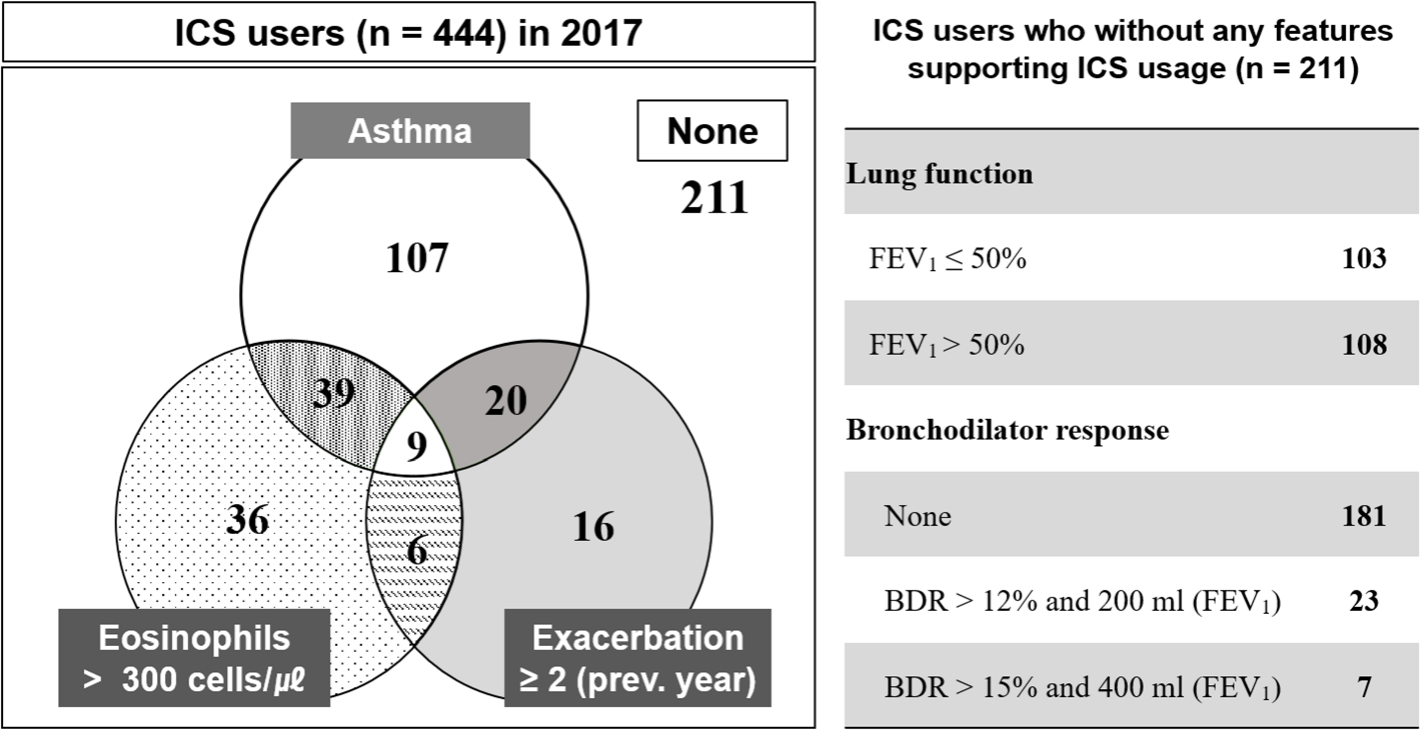
Venn diagram of features indicative of ICS usage among ICS users in 2017 Of the 444 ICS users in 2017, 175 (39.4%) had a history of asthma, 90 (20.2%) had blood eosinophilia, and 51 (11.5%) had experienced more than two exacerbations in the year prior to enrollment. Of the 211 (47.5%) patients who had no feature supporting ICS usage, 23 (10.9%) satisfied the bronchodilator response. BDR = bronchodilator response; FEV_1_ = forced expiratory volume in 1 s; ICS = Inhaled corticosteroid. ^†^*n* = 315

Of the 444 subjects using ICS-containing regimens in 2017, 175 (39.4%) had a history of asthma, 90 (20.2%) had blood eosinophilia, and 51 (11.5%) had experienced more than two exacerbations in the year prior to enrollment (Fig. 3). Of the 211 (47.5%) patients who did not exhibit any of these three features, a low number of subjects (10.9%) satisfied the bronchodilator reversibility criteria, while the majority (85.8%) did not show bronchodilator reversibility; and 103 patients (48.8%) had FEV_1_ of < 50%.

### ICS withdrawal and COPD exacerbation

The baseline characteristics of the ICS withdrawal and ICS continued groups were analyzed (Table 2); the significant differences between the groups were history of asthma and annual exacerbation frequency. More patients in the ICS withdrawal group were likely to have a history of asthma (56.6% vs. 41%, *p* = 0.0125); additionally, the ICS withdrawal group exhibited a higher annual exacerbation frequency than did the ICS continued group in the year prior to enrollment (0.79 vs. 0.53; *p* < 0.001). Patients were classified into two categories based on history of asthma and number of exacerbations in the year prior to enrollment (e-Table 2). In the ICS withdrawal group (*n* = 77), 45 patients (58.4%) were classified into ICS-continuation-preferred group; 31 patients (40.3%), into the ICS-withdrawal-preferred group.

**Table 2:** Comparison of the baseline characteristics of ICS withdrawal group and ICS continued group

	Withdrawal (n = 77)	Continued (n = 378)	p-value
Age	67.9 ± 8.3	68.4 ± 7.8	0.67
Male	74(96.1)	345(91.5)	0.24
Smoking status			0.72
Current smoker	27(35.1)	116(31.1)	
Ex-smoker	46(59.7)	231(61.9)	
Non-smoker	4(5.2)	26(7.0)	
Pack-years	43.7 ± 25.6	43.4 ± 24.9	0.93
BMI	23.1 ± 3.5	23.0 ± 3.3	0.47
SGRQ	34.0 ± 19.4	33.0 ± 18.6	0.65
CAT	15.9 ± 0.9	16.3 ± 0.4	0.90
mMRC	1.5 ± 0.9	1.6 ± 0.8	0.71
Post-BD FEV_1_, L	1.45 ± 0.44	1.48 ± 0.54	0.63
Post-BD FEV_1_, % predicted	54.0 ± 15.3	55.9 ± 19.9	0.44
Post-BD FEV_1_/FVC ratio	47.6 ± 11.0	47.9 ± 12.5	0.83
Bronchodilator Response			0.41
None	67(87)	307(81.2)	
BDR > 12% and 200 ml (FEV_1_)	7(9.1)	56(14.8)	
BDR > 15% and 400 ml (FEV_1_) (Spanish ACO)	3(3.9)	15 (4.0)	
DL_co_, % predicted^a^	70.2 ± 24.0	74.1 ± 23.8	0.24
Asthma	43 (56.6)	152 (41)	0.01
Exacerbation frequency, events/person-year	0.79 ± 2.35	0.53 ± 1.39	< 0.001
Serum eosinophils cells/μl^b^	177(84–325)	190(113–327)	0.23
Eosinophils > 300 cells/μl n (%)	14(25.5)	79(28.9)	0.6
GOLD group			0.39
A	32(42.7)	171(46.1)	
B	28(37.3)	134(36.1)	
C	8(10.7)	21(5.7)	
D	7(9.3)	45(12.1)	

Continuous data are presented as mean ± SD or median (interquartile range). Categorical variables as No. (%) unless otherwise indicated. See Table 1 legend for expansion of abbreviations. ^a^n = 382, ^b^n = 328

**Fig. 4:**
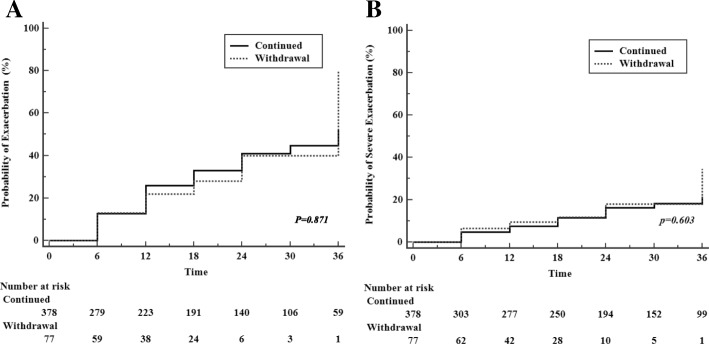
Kaplan-Meier plots of **a** time to first overall exacerbation **b** time to first severe exacerbation. There were no statistically significant differences between ICS withdrawal and continued groups in the time to first exacerbation or to first severe exacerbation. The *P* value for this trend was derived by using the Log-rank test

Times to the first exacerbation and to the first severe exacerbation were described by Kaplan-Meier graphs in Fig. 4a and b; neither of these indices were significantly different between ICS withdrawal and ICS continued groups.

**Fig. 5:**
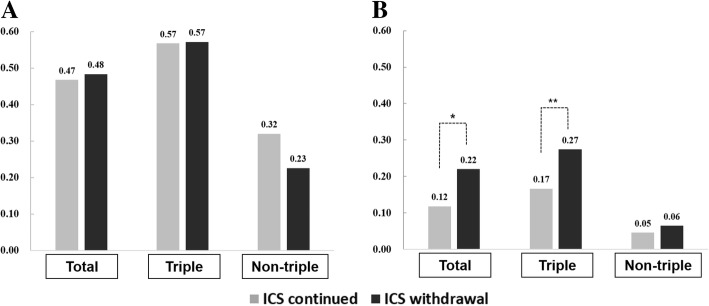
**a** Exacerbation and **b** Severe exacerbation rates per patient per year during the follow-up period **a** The annual rate of exacerbation was not significantly different between the ICS continued and withdrawal groups. **b** The annual rate of severe exacerbation in the ICS withdrawal group was statistically higher than that in the ICS continued group. * *P* = 0.031, relative risk (RR) 1.74 [95% confidence interval, CI: 1.05–2.88] ** *P* = 0.038, RR 1.80 [95% CI: 1.03–3.14]. ICS continued group (*n* = 378): patients with triple therapy (*n* = 234) and those with non-triple therapy (*n* = 144). ICS withdrawal group (*n* = 77): patients with triple therapy (*n* = 54) and with non-triple therapy (n = 23). ICS = inhaled corticosteroids

The annual exacerbation rate did not differ significantly between the ICS withdrawal and continued groups (Fig. 5a; 0.48 vs 0.47, *p* = 0.84). In both groups, the annual exacerbation rate significantly differed between the ICS triple therapy and non-triple therapy groups (continued group, 0.57 vs. 0.32, *p* < 0.001; withdrawal group, 0.57 vs. 0.23, *p* = 0.017). The annual rate of severe exacerbation in the ICS withdrawal group was statistically higher than that in the ICS continued group (Fig. 5b; 0.22 vs. 0.12, *p* = 0.031; Relative risk (RR) 1.74 [95% confidence interval, CI: 1.05–2.88]). The difference in annual severe exacerbation rates was more prominent in triple-therapy users (0.27 vs. 0.17, *p* = 0.038, RR 1.80 [95% CI: 1.03–3.14]). When the severe exacerbation rate was adjusted for confounders, ICS withdrawal did not significantly impact severe exacerbation frequency (*p* = 0.082, RR 1.623 [95% CI: 0.94–2.82]).

## Discussion

The guidelines for the treatment of stable COPD changed substantially in 2017 [24]. A fixed combination of LAMA/LABA was approved as first-line therapy, and ICS was recommended as second-line treatment, or for the treatment of specific conditions [24, 25, 31]. ICS-containing regimens were prescribed in approximately 50% of patients at baseline; this decreased to 38.8% in 2107, and ICS-containing regimen were replaced by LAMA/LABA combination therapy. Although ICS withdrawal was not associated with an increased risk of overall exacerbation, severe exacerbation was higher in the ICS withdrawal group. Further, more participants had a history of asthma and exacerbation in the ICS withdrawal group than in the ICS continuation group at baseline; this may have impacted the results.

ICS was previously approved as a first-line therapy for COPD patients with severe airflow limitation and frequent exacerbations and was used to reduce symptoms in patients with COPD [9]. Our study found that ICS users exhibited lower FEV_1_ and more frequent exacerbations at baseline than ICS nonusers. Additionally, the ICS-user group included a higher proportion of patients with bronchodilator response, severe symptoms, and poor quality of life relative to the ICS-nonuser group. As prescription of ICS was ultimately determined by the physician, these differences in baseline characteristics likely reflect the criteria used for ICS prescriptions in clinical practice and can be attributed to consideration of the benefit of ICS for attenuation of a patient’s particular manifestation of COPD.

The indication of ICS is an important issue in COPD management. ICS is currently recommended for patients with ACO, or with frequent exacerbations despite the use of long-acting bronchodilators [24, 25]. When the diagnosis of asthma is unclear, the presence of a significant bronchodilator response and/or significant blood eosinophilia (> 300 cells/μl) are regarded as features highly suggestive of ACO [30]. Blood eosinophilia is a well-documented factor predictive of ICS responsiveness [31, 32]. With these considerations, this study adopted a a history of asthma, eosinophilia, and frequent exacerbations as indicators for ICS usage. Surprisingly, approximately half (47.5%) of ICS users in 2017 had none of these features. Clinicians might have continued ICS on account of patients having presented severe symptoms or airflow limitation, although they alone are not indications for the use of ICS. Furthermore, patients were found to have only one feature supporting ICS usage. Sputum or blood eosinophilia is a reasonable biomarker for frequent exacerbations [31, 33, 34], indicating that features indicative ICS usage are interrelated. Our study showed that these features overlapped in a relatively small proportion of patients; therefore, these features should be checked to identify patients who may benefit from ICS therapy.

Significantly higher rates of severe exacerbation were found in the ICS withdrawal group than the ICS continued group. However, patients in the former showed greater exacerbation frequency at baseline (Table 2). This suggested that patients for whom the benefits of ICS use were greater than the risks might have unreasonably discontinued ICS, thus increasing the number of exacerbation events. Although the specific reasons for ICS withdrawal were not identified, the side effects of ICS or pneumonia might have influenced this decision. This also indicated that ICS withdrawal should be considered from various perspectives.

Many studies analyzed the effect of ICS withdrawal on lung function, exacerbation rate and adverse events among the different subset of COPD populations [35–38]. ICS withdrawal generally did not increase overall exacerbation: but, patients with eosinophilia showed a deleterious effect after ICS withdrawal in post-hoc analysis [37, 38]. Eosinophil was also documented as a useful indicator to choose initial therapy in the recent real-world observational study [39]. In this study, it is not easy to show the difference in exacerbation by eosinophil count due to relatively small number of patients. Meanwhile, 46.4% of patients with eosinophilia were ICS users and only 20.2% of ICS users had eosinophilia. This result reflects blood eosinophilia itself has not been widely used as a sole indicator for ICS usage in real practice. Blood eosinophil level would be considered more in withdrawal and initiation of ICS.

This study features the following merit. First, our analysis included three prospective COPD cohorts, which together account for more than 30 hospitals; our findings therefore reflects real-world data from heterogeneous COPD populations in one country. Second, these cohorts has a relatively long follow-up period: In the ICS withdrawal group, patients had been using ICS for an approximate mean of 21.8 months and the mean follow-up period after withdrawal was 17.8 months. This long follow-up period allowed for the sufficient evaluation of exacerbation.

The present study is subject to several limitations. First, unlike previous studies, we did not exclude patients with history of asthma; the result of our study should thus be interpreted carefully. Second, due to the observational nature of our study design, small number of patients (16%) underwent ICS withdrawal during follow up period, causing a discrepancy between the sample sizes of the ICS withdrawal and continued groups. This discrepancy might bias understanding of the exacerbation analysis. The reasons for withdrawal of ICS at that time could not be identified. We were unable to adjust the model according to each different type of inhalers. Lastly, as majority of the patients were men with a history of smoking, it is difficult to generalize the result form this study to non-smokers or women with of COPD.

## Conclusions

The introduction of fixed dual long-acting bronchodilator therapy has changed the pattern of inhaler prescriptions for patients with COPD: prescriptions of ICS are rapidly being replaced by those of LAMA/LABA. Our results suggest that ICS withdrawal without sufficient consideration of the patient’s clinical features might impact exacerbation. ICS withdrawal should therefore be weighed from various perspectives with an appropriate evaluation of the attendant risks and benefits.

## Abbreviations

ACO: Asthma and COPD overlap
BD: Bronchodilator
COPD: Chronic obstructive pulmonary disease
FEV_1_: Forced expiratory volume in 1 s
FVC: Forced vital capacity
GOLD: Global Initiative for Chronic Obstructive Lung Disease
ICS: Inhaled corticosteroid
LABA: Long acting beta2 agonist
LAMA: Long acting muscarinic antagonist
